# Single-cell dataset of juvenile mouse neurons from the thalamic reticular nucleus: whole-cell patch-clamp electrophysiology

**DOI:** 10.3389/fnins.2026.1927631

**Published:** 2026-10-29

**Authors:** Maurizio Pezzoli, Rodrigo Perin, Henry Markram

**Affiliations:** 1 Laboratory of Neural Microcircuitry (LNMC), Brain Mind Institute, École Polytechnique Fédérale de Lausanne (EPFL), Lausanne, Switzerland; 2 Blue Brain Project (BBP), Brain Mind Institute, École Polytechnique Fédérale de Lausanne (EPFL), Lausanne, Switzerland

**Keywords:** icephys, *in vitro* slice, mouse, electrophysiology, whole-cell current-clamp, dataset, thalamic reticular nucleus

## Abstract

This dataset provides single-cell whole-cell patch-clamp electrophysiology for 19 interneurons of the thalamic reticular nucleus (TRN) in juvenile mice. The recordings were obtained from male C57Bl/6J mice sampled within the developmental window of postnatal days 24 to 34. Acute horizontal brain slices were used, and visually targeted whole-cell recordings were performed in current-clamp mode. A standardized current-clamp stimulus battery was used identically across all cells to characterize their intrinsic electrophysiological properties—passive membrane behavior, action-potential threshold and waveform, suprathreshold firing patterns, and the hyperpolarization-evoked rebound-burst dynamics characteristic of reticular thalamic neurons. The release comprises 18,015 recorded sweeps and their full, un-decimated signal traces (400 million digitized samples), preserved in the community-standard NWB (icephys) format alongside the original Igor Pro source files, and indexed into tidy, queryable Apache Parquet tables—one record per recording session, one per sweep, and one per digitized sample—linked by a normalized recording identifier. Each recording includes animal metadata (postnatal age, body weight, strain, genotype, slice orientation, and recorded region) and its resting membrane potential. The dataset is released as a FAIR² Data Package under the Open Data Commons Attribution License v1.0 (ODC-By v1.0), with machine-readable metadata conforming to the MLCommons Croissant specification, QUDT units, and PROV-O provenance. It is intended for neuroscientists, computational modelers, and machine-learning researchers studying the intrinsic electrophysiology of TRN neurons.

## Introduction

Comprehending the intrinsic electrophysiological behavior of neurons in the thalamic reticular nucleus (TRN) is central to understanding how the thalamus regulates the flow of sensory information to the cortex (Crandall et al., 2015; Halassa and Acsády, 2016). The TRN is a thin shell of GABAergic neurons that surrounds the dorsal thalamus and provides the principal source of inhibition to thalamocortical relay cells; its intrinsic firing repertoire—particularly the rebound low-threshold calcium bursts that follow membrane hyperpolarization (Halassa and Acsády, 2016)—shapes the rhythms of sleep and arousal and has been implicated in absence epilepsy and disorders of attention. Characterizing these cells functionally requires whole-cell patch-clamp recordings acquired under a standardized, reproducible stimulus battery, so that passive membrane properties, action-potential generation, and firing behavior can be compared consistently across cells.

This dataset addresses the need for research on TRN neurons, as studied at the EPFL Laboratory of Neural Microcircuitry (LNMC), and it was gathered by Maurizio Pezzoli (MPG). It includes a library of standardized whole-cell patch-clamp recordings from 19 identified TRN neurons of juvenile C57Bl/6J mice, recovered and harmonized from the original LNMC laboratory archives. A standardized current-clamp stimulus battery was applied across all recorded cells, enabling a consistent comparison of intrinsic membrane properties, action-potential generation, and firing behavior among the TRN neurons of juvenile mice.

The advent of machine learning in neuroscience has raised the bar for data reuse: training pipelines require curated, well-documented, standardized resources with machine-readable provenance, computable licenses, and unambiguous dataset boundaries. To meet these needs, the recordings are preserved in the community-standard (Rübel et al., 2022) NWB (icephys) format alongside their original Igor Pro source files, and are indexed into tidy, queryable Parquet tables—one row per recording session, one per sweep, and one per digitized sample—linked by a normalized recording identifier.

The result is provided as a FAIR² Data Package (Wilkinson et al., 2016)—Findable, Accessible, Interoperable, Reusable, AI-ready, and Responsibly governed—with structured metadata conforming to the MLCommons Croissant specification (MLCommons, 2024), QUDT units, and PROV-O provenance. This article documents the dataset's design, contents, methods, and quality, and provides a visual overview of the electrophysiological data.

## Methods

### Study design

This study characterizes the intrinsic electrophysiological properties of TRN neurons in juvenile male mice. Experiments were conducted on wild-type mice (C57Bl/6J) sampled within postnatal days 24 to 34 (P24–P34), following the extraction of *ex vivo*, acute brain slices that contained TRN neurons. Each recorded neuron was acquired in a single integrated whole-cell patch-clamp experiment, providing an electrophysiological fingerprint. Each recording was characterized by a quantitative electrophysiological descriptor covering passive membrane properties, action-potential shape, and firing dynamics.

### Animals

Experimental subjects were juvenile wild-type male mice (C57Bl/6J), post-natal 24 to 34 days old (P24-P34). This developmental window was selected because the thalamic microcircuits were sufficiently mature to be visually identifiable and to allow stable patch-clamp recording, while tissue viability during acute slicing remains high. All animal procedures were conducted in accordance with Swiss national legislation and the institutional guidelines of the École Polytechnique Fédérale de Lausanne (EPFL). The animals were anesthetized with Isoflurane 4.5% and rapidly decapitated. The brain was carefully removed and immediately placed in ice-cold cutting artificial cerebrospinal fluid (cACSF) to minimize post-mortem deterioration of the tissue prior to sectioning.

### Slice preparation

Acute horizontal slices of 300 μm thickness were cut from each brain on a Leica VT 1200S vibratome (Leica Biosystems, USA). The slices contained sections of the TRN. The sectioning was performed in oxygenated (O_2_ 95%, CO_2_ 5 % mix) ice-cold cutting artificial cerebrospinal fluid (cACSF). Following sectioning, the slices were left to incubate in oxygenated (O_2_ 95%, CO_2_ 5 % mix) ACSF for 1 h at room temperature, and they remained in this solution until the time of recording. For recording, individual slices were transferred to a chamber perfused with oxygenated ACSF and held at 33 °C ± 1 °C. Neurons from the TRN were visualized by infrared differential interference contrast (IR-DIC) video microscopy, using a Prime 95B sCMOS camera (Teledyne Photometrics, USA) mounted on an upright BX51WI microscope (Olympus, Japan), and were targeted on the basis of their somatic position.

### Solutions

Slices, while being cut, were continuously superfused with ice-cold cutting artificial cerebrospinal fluid (cACSF) containing213 Sucrose, 25 NaHCO_3_, 2.5 KCl, 1.25 NaH_2_PO_4_, 0.5 CaCl_2_, 10 MgCl_2_, and 25 D-glucose, in millimolar concentrations, bubbled with a mixture of 95% O_2_ and 5% CO_2_. After sectioning, the slices were incubated at room temperature, continuously superfused with artificial cerebrospinal fluid (ACSF) containing, in millimolar concentrations, 125 NaCl, 25 NaHCO_3_, 2.5 KCl, 1.25 NaH_2_PO_4_, 2 CaCl_2_, 1 MgCl_2_, and 25 D-glucose, bubbled with a mixture of 95% O_2_ and 5% CO_2_. The carbogen-equilibrated ACSF was maintained around the slices throughout recordings. Unless otherwise specified, chemicals were obtained from Sigma-Aldrich (St. Louis, MO) or Merck (Darmstadt, Germany). The intracellular pipette solution contained 110 K-gluconate, 10 KCl, 4 ATP-Mg, 10 phosphocreatine, 0.3 GTP, 10 HEPES, and 8 biocytin, in millimolar concentrations. The solution was adjusted to pH 7.3–7.4 using 5 M KOH, and osmolarity was adjusted to 290–300 mOsm using D-mannitol at 25–35 mM. Membrane potential values were not corrected for the liquid junction potential, which was approximately −14 mV.

### Electrophysiological recordings

Patch pipettes were fabricated from borosilicate glass using a Flaming/Brown micropipette puller, model P-97 (Sutter Instruments, Novato, CA), and were pulled to tip resistances of 5 to 10 MΩ. Pipettes were filled with the intracellular solution and used to establish whole-cell patch-clamp recording on visually targeted neurons. Single-cell somatic recordings were performed in current-clamp mode using Axopatch 200B amplifiers (Molecular Devices, San Jose, CA), with the recording temperature maintained at 33 °C ± 1 °C. Series resistance and seal stability were monitored continuously throughout the recording. Data acquisition was performed via an ITC-1600 board (Instrutech, Port Washington, NY) connected to a computer running custom routines under IgorPro (WaveMetrics, Portland, OR). Signals were low-pass filtered at 2 kHz using a Bessel filter and sampled at 10 and 50 kHz, defining the temporal resolution and bandwidth of every recorded trace. To avoid any possible unwantedly induced high-frequency noise in the current-clamp recordings, the capacitance was not compensated. Neurons were bridge-balanced. Only recordings with access resistance < 20 MΩ throughout the full protocol were retained.

### Stimulus protocol

Each cell was subjected to a standardized current-clamp stimulus battery consisting of distinct stimulus protocols, which were applied identically across all recordings. The battery covers electrode and recording-stability checks, subthreshold passive properties, hyperpolarization/afterhyperpolarization behavior, action-potential threshold and waveform, suprathreshold firing patterns, spike-recovery and refractoriness, and resonance/noise characterization (Table 1). This ensures that the resulting quantitative electrophysiological descriptor is comparable across cells.

**Table 1:** Stimulus protocol description.

Stimulus name	Protocol description	Features	Number of sweeps per stimulus	Number of repetitions per stimulus	Current or voltage clamp
APWaveform	Incremental current steps (6×) at high sampling rate (50 Hz) at threshold voltage values	AP threshold AP amplitude AP width General AP analysis	6	1	CC
DelHyperPol	Incremental hyperpolarizing current steps (4×) followed by a depolarizing current step to suprathreshold voltage values	Voltage-activated and tail currents at hyperpolarizing transitions to subthreshold values	4	1	CC
Delta	Discrete hyperpolarizing current pulse	Passive membrane properties at subthreshold values (e.g., voltage relaxation time constant)	1	30	CC
ElecCal	Fixed injected current noise	Subthreshold frequency analysis	1	3	CC
FirePattern	Long (4 s) incremental current steps (2×) at threshold voltage values	Inter-AP intervals AP firing frequency AP firing pattern General AP analysis	2	1	CC
HyperDePol	Incremental hyperpolarizing current steps (7×) followed by a depolarizing current step at threshold voltage values	Voltage-activated and tail currents at depolarizing transitions to threshold	7	1	CC
HyperDePolBurst	Incremental hyperpolarizing current steps (6×) followed by a return to holding current	Voltage-activated and tail currents at depolarizing transitions to holding current values	6	1	CC
IDRest	Incremental depolarizing current steps (11×) at threshold and suprathreshold voltage values	AP threshold Inter-AP intervals AP firing frequency AP firing pattern General AP analysis	11	1	CC
IDThres	Small incremental depolarizing current steps (21×) at threshold voltage values	AP threshold General AP analysis	21	1	CC
IV	Depolarizing and hyperpolarizing current steps (11×) at subthreshold voltage values	Membrane input resistance	11	1	CC
IVRest	Depolarizing and hyperpolarizing current steps (11×), at high sampling rate (50 Hz), at subthreshold, threshold and suprathreshold voltage values	Membrane input resistance AP threshold Inter-AP intervals AP firing frequency AP firing pattern General AP analysis	22	1 (at end of all protocols)	CC
NoiseOU3	Fixed injected current noise	Subthreshold frequency analysis	1	1 (at end of all protocols)	CC
PosCheops	Continuous trace with depolarizing and hyperpolarizing ramps of current injection, with 3 different slopes, between subthreshold (holding current) and suprathreshold voltage values	AP threshold General AP analysis	1	1	CC
RPip	Voltage step ± 5 mV	Pipette input resistance	1	Variable	VC (0 mV)
RSealClosed	Voltage step ± 5 mV	Seal input resistance	1	Variable	VC (−70 mV)
RSealOpen	Voltage step ± 5 mV	Membrane input resistance	1	Variable	VC (−70 mV)
Rac	Hyperpolarizing current pulse at high sampling rate (50 Hz)	Inner testing to calibrate access resistance	1	Variable	CC
ResetITC	Fixed injected current white noise	Inner testing checkpoint Subthreshold frequency analysis	1	1	CC
SetAmpl	Depolarizing current pulse	Inner testing checkpoint to reach threshold values	1	Variable	CC
SinSpec	Injected sinusoidal current wave (3×) of increasing frequency (0–250 Hz)	Subthreshold frequency analysis	1	3	CC
SpontHold3	Short (3 s) holding current injection	Holding current for −70 mV ± 5 mV	1	1	CC
SpontHold30	Long (10 s) holding current injection	Holding current for −70 mV ± 5 mV Membrane voltage stability	1	1	CC
SpontNoHold30	Long (10 s) with no holding current	Resting membrane potential (current zero values) Membrane voltage stability	1	1	CC
StartHold	Very short (100 ms) holding current injection	Inner testing checkpoint Holding current for −70 mV ± 5 mV	1	1	CC
StartNoHold	Very short (100 ms) with no holding current	Inner testing checkpoint Resting membrane potential	1	1 (at the beginning of full protocol recording per neuron)	CC
TestAmpl	Depolarizing current step at the vicinity of threshold values	Inner testing checkpoint for threshold values	1	Variable	CC
TestRheo	Incremental depolarizing current steps (6×) at threshold voltage values	AP threshold Rheobase	6	1	CC
sAHP	Incremental depolarizing current steps (4×) at suprathreshold voltage values, followed by a transition to threshold voltage value	Voltage-activated and tail currents at hyperpolarizing transitions to threshold values General AP analysis	4	1	CC

A standardized set of current injection protocols to scan voltage responses. Except for those used for internal testing, or for consistency check, all stimulus sets were aimed to be obtained in triplicate per cell. Curation criteria kept recordings that present at least two stable recordings per stimulus protocol. Resting membrane potential was kept at −70 mV ± 5 mV, with a holding current if necessary. Between sweeps, the neuron returns to the holding current value for standardization. For each stimulus sweep, the injected current would be the holding current plus the stimulus. Many sweeps in one stimulus indicate that the same is repeated but with an incremental current delta on each new sweep. Repetitions indicate the number of recordings performed in holding current plus stimulus per protocol. The features presented are either properties measured directly or can be obtained by simple mathematical analysis (e.g., membrane input resistance) from the full set of protocols. They range among those describing action potentials (AP), firing patterns, subthreshold voltage-activated current dynamics, as well as passive membrane properties.

## Data overview

### Dataset content and curation

The dataset contains an acute sample of TRN neurons from a group of juvenile male (p24-34) wild-type (C57Bl/6J) mice. The age range was selected to be functionally mature for the thalamocortical circuit, while also providing good-quality *ex vivo* slices. The dataset provides stable, clean recordings of 19 TRN neurons in a comparable manner. The anatomical distribution of the recorded neurons along the dorsoventral axis ranged from −3.16 to −4.12 mm from the bregma (Paxinos and Franklin, 2019). This places the neurons along the first-order thalamus (motor and somatosensory). The full protocol was a battery of different current-clamp stimulations, at least in duplicate in each neuron, to explore all possible range of voltage responses within a physiologically relevant range. The aim was to provide tests that uncover electrophysiological passive and active properties. Each voltage (V) recording trace carries a sister current trace (A) which shows the current values corresponding to the holding current injection plus the current applied for the stimulus (identified by the same sweep number in the trace file name), except for RPip, RSealOpen, and RSealClosed traces, which were performed in voltage-clamp conditions.

Applied quality criteria retained only recordings from neurons with the exact same protocol set. To standardize the recordings, voltage must be maintained stable between stimuli at −70 mV ±5 mV during the full set. If necessary, initially a steady holding current was injected to adjust the voltage to that value. Only recordings that had access resistance < 20 MΩ during the full protocol were retained. Except for a few test or closing stimuli (e.g., IVRest), each set was aimed to be a triplicate for each cell to provide internal consistency. The data presented here are from neurons in which at least 2 full, stable protocol sets were recorded successfully.

### Quantitative summary of the dataset

This dataset provides single-cell whole-cell patch-clamp electrophysiology for TRN neurons of juvenile mice. It is assembled as a FAIR² package (Wilkinson et al., 2016) with three tidy tables—one record per recording session, one record per sweep, and one record per digitized sample—linked by a normalized Igor Pro experiment identifier. Across the package, the dataset spans 19 recording sessions (19 identified TRN neurons from 13 animals), 18,015 recorded sweeps, and 399,619,819 digitized signal samples. The canonical raw signals are preserved as 19 NWB (HDF5) files (one per neuron, ~1.5 GB) and as the 19 original Igor Pro.h5xp source files (~1.9 GB).

### Dataset composition

The package comprises three canonical asset groups linked by the Igor Pro experiment identifier (igor_file_name):

- Recording sessions: A total of 19 recordings carried in mouse_metadata (19 rows × 15 columns)—one row per neuron/recording session, holding animal identity, sex, strain, genotype, postnatal age, body weight, project, slice orientation and coordinate, recorded region, resting membrane potential, and the Igor Pro file stem that keys the sweep tables.
- Sweeps: 18,015 recorded sweeps in sweep_metadata (18,015 rows × 19 columns)—one row per Igor Pro wave, capturing the stimulus protocol name, ADC/DAC channel, sweep index, sample count, sampling interval and rate, sweep duration, signal units, clamp mode, wave-note recording date/time, and the full protocol definition string. Recordings hold a median of 912 sweeps each (range 658–1,494).
- Trace samples: 399,619,819 digitized samples in sweep_traces (399,619,819 rows × 5 columns)—one row per sample, with the sweep and channel keys, the zero-based sample index, the time in seconds relative to sweep onset, and the recorded signal value (voltage in V for current-clamp sweeps; current in A for the paired command channel). This is the full, un-decimated time series for every sweep across all 19 neurons.

The 19 original Igor Pro.h5xp files are preserved in assets/h5xp/, and 19 NWB (HDF5) files—1 per neuron—are provided in assets/nwb/.

### Data structure and formats

The package follows the standard FAIR² wrangler layout: canonical scientific data as NWB:N icephys (HDF5) in assets/nwb/, the original Igor Pro.h5xp sources in assets/h5xp/, and a tabular projection as three ZSTD-compressed Apache Parquet tables in data/parquet/ (mouse_metadata, sweep_metadata, sweep_traces). Machine-readable metadata is provided in fair2.json (MLCommons Croissant 1.1 + W3C PROV-O provenance) and data_dictionary.csv (a variable-level catalog of every column with type, unit, description, and ontology concept). The tables join on igor_file_name (mouse_metadata ↔ sweep_metadata ↔ sweep_traces) and on wave_name (sweep_metadata ↔ sweep_traces). Extraction scripts in scripts/ (extract_mouse_metadata.py, extract_h5xp.py, convert_h5xp_to_nwb.py, and helpers) document exactly how the source waves were transformed.

### Feature and attribute space

Per-recording metadata exposes animal identity and husbandry attributes (animal_id, sex, strain, genotype, date_of_birth, age_days, body_weight_g), acquisition context (project, slice_cut_orientation, slice_coordinate_mm, neuron_recorded, date_of_dissection, license), and a single physiological readout at session start (initial_vm_mv, the resting membrane potential at zero current injection). Per-sweep metadata exposes the stimulus identity and acquisition parameters (stimulus_name, channel, sweep_index, n_samples, dt_s, sampling_rate_hz, duration_s, signal_units, ADC/DAC channel descriptors, and the wave-note timestamp and protocol_def). The trace table exposes the raw signal itself (time_s, value). This release distributes the raw recorded signal and its acquisition metadata; a derived quantitative electrophysiological feature table (e-features) is not part of this package.

### Categorical and taxonomic diversity

- Animals: juvenile (p24-p34) male mouse (*Mus musculus*, C57Bl/6J, wild type). All 19 recordings are drawn from 13 individuals.
- Recorded region: TRN—annotated TRN in all recordings.
- Slice orientation: horizontal in all recordings.
- Stimulus protocols (28 distinct “stimulus_name” values across 18,015 sweeps): a standardized current-clamp battery covering electrode/recording-stability and housekeeping checks (e.g., ElecCal, SetAmpl, TestAmpl, ResetITC, StartHold), spontaneous/holding acquisitions (SponHold3, SponHold30, SponNoHold30), subthreshold passive and current–voltage probes (IV, IVRest, TestRheo, Delta, Rac), hyperpolarization/afterhyperpolarization behavior (HyperDePol, HyperDePolBurst, DeHyperPol, sAHP), action-potential threshold and waveform (IDThres, APWaveform, PosCheops), suprathreshold firing patterns and adaptation (IDRest, FirePattern, IVRest), and resonance/noise characterization (SineSpec, NoiseOU3, ElecCal, ResetITC). More detailed information on stimulus protocols is given in Table 1. The 10 most-represented protocols by sweep count are Delta (3,480), IDThres (2,470), Rac (1,841), IV (1,320), TestRheo (1,452), IDRest (946), HyperDePol (802), APWaveform (726), IVRest (790), and HyperDePolBurst (698).

### Data completeness

- Metadata: all 19 recordings carry complete animal identity, age, weight, slice orientation, and dorsoventral coordinate from bregma in mm, recorded region, and initial resting membrane potential; the initial resting membrane potential spans −83 to −55 mV (mean −68.7 mV). TRN neurons recorded were distributed along the dorsoventral (D.V.) axis from the bregma point, in the range of −3.16 mm to −4.12 mm, locating them by first-order thalamus.
- Electrophysiology: every recording carries the standardized stimulus battery (median 912 sweeps per recording, range 658–1,494), and each sweep is stored as its complete, un-decimated sample series in sweep_traces (399,619,819 samples in total).
- Sampling: sweeps were acquired at one of two rates, and the rate is a property of the stimulus protocol rather than of the recording—every protocol was acquired at a single rate, and all 19 recordings contain both. Twenty-five protocols were acquired at 10 kHz (14,658 sweeps); three were acquired at 50 kHz (3,357 sweeps): Rac (1,841), IVRest (790), and APWaveform (726). The per-sweep rate is carried in sweep_metadata.sampling_rate_hz.
- No imputation or interpolation was applied; the raw signals are preserved in the canonical NWB and.h5xp files, and the Parquet sweep_traces table is a faithful, full-resolution projection of the recorded waves.

### Notable patterns

- All 19 recordings are current-clamp whole-cell recordings of male juvenile mouse TRN neurons of first-order thalamus within a narrow developmental window (postnatal day 24–34, median 26 days), giving a demographically homogeneous cohort.
- The recorded signal is carried on the voltage channel (membrane potential, resting near −70 mV in the passive regime) with the paired command-current channel stored alongside it, so both the response and the injected stimulus are recoverable per sweep.
- The most heavily sampled protocols are the suprathreshold and threshold-crossing families (Delta, IDThres, IDRest, APWaveform) together with the subthreshold current–voltage and afterhyperpolarization probes (IV, TestRheo, HyperDePol, sAHP), consistent with a battery designed to characterize the TRN firing repertoire—passive properties, spike threshold and shape, and the rebound-burst behavior that follows hyperpolarization.

## FAIR² compliance certification

### Overall FAIR² badge assessment

Compliant—the dataset meets the FAIR² criteria (Wilkinson et al., 2016). Findability is established through a registered DOI (10.71728/senscience.k7f3-9xqm) and a structured fair2.json carrying Croissant 1.1 metadata and a PROV-O provenance graph. Accessibility is provided through the canonical NWB (HDF5) distribution, the preserved Igor Pro.h5xp sources, and the tidy Parquet projection. Interoperability is supported by community-standard formats (NWB:N icephys for electrophysiology, Apache Parquet for tabular data, JSON-LD for metadata) and QUDT units. Reusability is supported by a variable-level data dictionary, field-level PROV-O provenance to the extraction scripts, and an explicit ODC-By v1.0 license (Open Data Commons, 2017). An automated integrity assessment scored 0.98. The FAIR^2^ assessment criteria and supporting evidence are summarized in Table 2.

**Table 2:** FAIR^2^ compliance assessment criteria and supporting evidence for the curated multimodal neuronal dataset.

Criteria	Details
Findability (F)	
F1. Unique identifier	The dataset is assigned a DOI (10.71728/senscience.k7f3-9xqm) registered with DataCite for global traceability and citability.
F2. Metadata	Metadata includes title, creators and affiliation (EPFL LNMC/BBP), abstract, species (*Mus musculus*, C57Bl/6J), brain region (TRN), variables across three tables, and the data-collection and extraction methods.
F3. Metadata includes data identifiers	The DOI (10.71728/senscience.k7f3-9xqm) is embedded in the metadata; table and asset identifiers (Igor Pro file stems and wave names) link the metadata record to the distributed files.
F4. Searchable Metadata	Metadata is exposed as schema.org/Dataset (JSON-LD) and as an MLCommons Croissant 1.1 record, indexable by Google Dataset Search.
Accessibility (A)	
A1. Open access	Released under the Open Data Commons Attribution License v1.0 (ODC-By v1.0) license, permitting reuse, redistribution, and modification with attribution (Open Data Commons, 2017).
A2. Long-term access	Canonical NWB assets and the original Igor Pro.h5xp sources are preserved within the package alongside the derived tables (Rübel et al., 2022).
API access	Structured metadata and downloadable components are exposed via the MLCommons mlcroissant API and the FAIR² portal data explorer (MLCommons, 2024; Wilkinson et al., 2016).
Interoperability (I)	
I1. Standardized formats	Tabular data in Parquet; electrophysiology in NWB:N icephys (HDF5) with original Igor Pro.h5xp sources; semantic metadata in JSON-LD.
I2. Controlled vocabularies	Units standardized with QUDT. Fields annotated with mappings to schema.org, NCIT, UBERON, and PATO. Metadata schema based on schema.org, Croissant, and PROV-O.
I3. Cross-platform integration	Conforms to MLCommons Croissant 1.1 for AI workflows; NWB is the community standard for the neurophysiology domain.
Reusability (R)	
R1. Comprehensive documentation	A variable-level data dictionary describes every column across the three tables with definitions, units, and method provenance.
R1.1. License	ODC-By v1.0, permitting reuse, redistribution, and modification with attribution.
R1.2. Detailed provenance	Each table and field is linked via PROV-O to the extraction scripts (extract_mouse_metadata.py, extract_h5xp.py, convert_h5xp_to_nwb.py) and the source.h5xp archives.
R1.3. Domain-relevant standards	Electrophysiology follows the NWB:N icephys standard; recordings are current-clamp whole-cell acquisitions.
Versioning and updates	Version 1.0.0 with embedded version metadata.
AI-Readiness (AIR)	
Structured for machine learning	Tidy tabular Parquet with consistent identifiers and a session↔sweep↔sample join key, suitable for ML ingestion.
Scalable	~400 million trace samples across 18,015 sweeps, indexed for query while raw arrays remain in the canonical NWB/.h5xp files.
Training and validation sets	Natural partitioning by recording session and by stimulus protocol supports train/validation/test splits.
Responsible AI (RAI)	
Ethical standards and misuse	Intended for neuroscience research on TRN neuron intrinsic electrophysiology.
Biases in the dataset	All subjects are male juvenile C57Bl/6J mice; coverage reflects the LNMC project MPG sampling and is not a population-representative survey.
Data privacy and security	No human personal data. All subjects are non-human animals (*Mus musculus*); the privacy screening decision is ALLOW_PUBLIC. Contributor names appear in a standard scientific-attribution context.
Data provenance and accountability	Field-level provenance is encoded with PROV-O and documented in the data dictionary.
Transparency and reporting	Extraction methods and known limitations are documented; an automated integrity assessment scored 0.98.
Human-in-the-Loop (HITL) considerations	Source recordings were produced by the LNMC; FAIR² packaging was performed by an automated wrangling agent with documented steps.

### Visual overview

The following figures provide a visual overview of the dataset—the composition of the recorded cohort, the standardized stimulus battery, representative whole-cell current-clamp traces, and the composition of the recordings themselves.

The composition of the recorded cohort is characterized in Figure 1, summarizing the demographic and physiological range of the 19 recorded cells.

**Figure 1:**
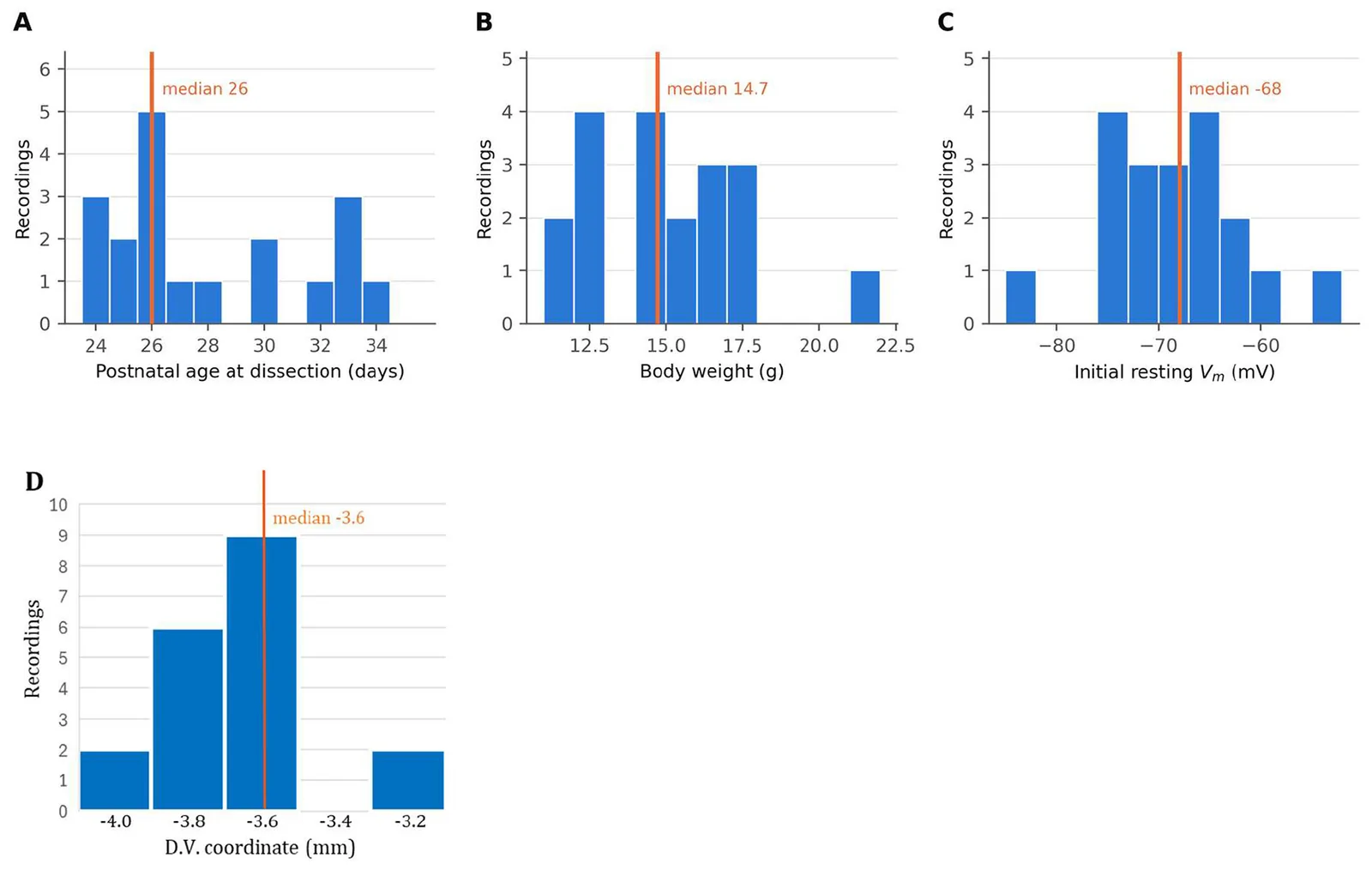
Cohort composition across the 19 recording sessions. **(A)** Distribution of postnatal age at dissection (range 24–34 days; median 26). **(B)** Distribution of body weight (11.8–21.8 g). **(C)** Distribution of the initial resting membrane potential recorded at session start (−83 to −55 mV; mean −68.7 mV). **(D)** Distribution of the recorded neurons along the dorsoventral axis (−3.16 to −4.12 mm), from the bregma point (Paxinos and Franklin, 2019). All animals are male C57Bl/6J wild-type mice.

The standardized stimulus battery is summarized in Figure 2, showing how sweeps are distributed across protocols.

**Figure 2:**
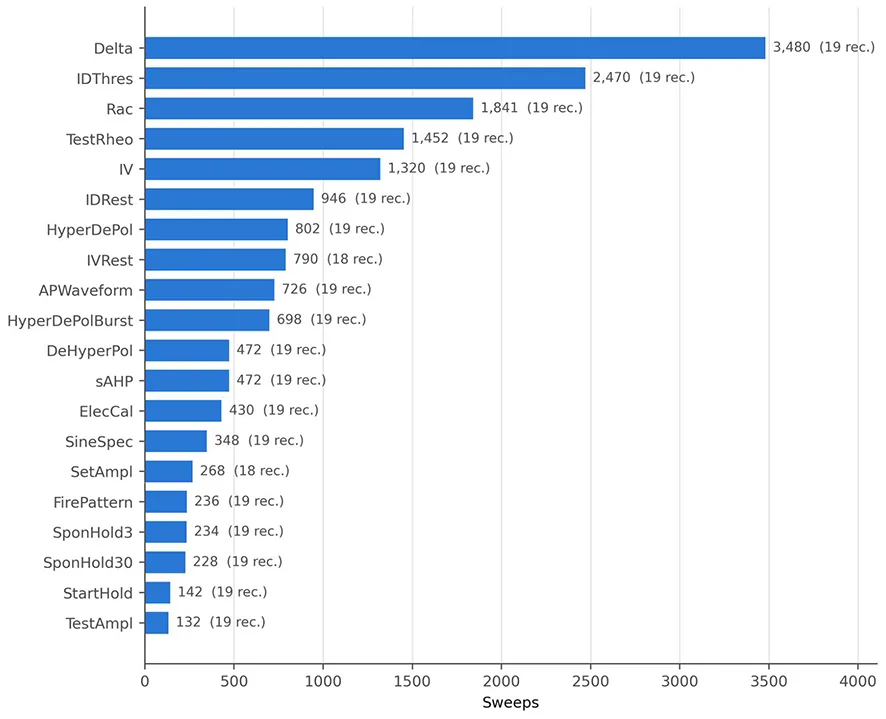
The standardized current-clamp stimulus battery. The 20 most-represented of the 28 distinct stimulus protocols are shown by total sweep count, with the number of recordings contributing to each protocol annotated. The battery spans electrode/stability checks, subthreshold current–voltage probes, hyperpolarization/afterhyperpolarization behavior, action-potential threshold and waveform, suprathreshold firing patterns, and resonance/noise stimuli.

The voltage responses from a single cell are shown in Figure 3, illustrating the diversity of intrinsic behaviors probed by the standardized stimulus battery.

**Figure 3:**
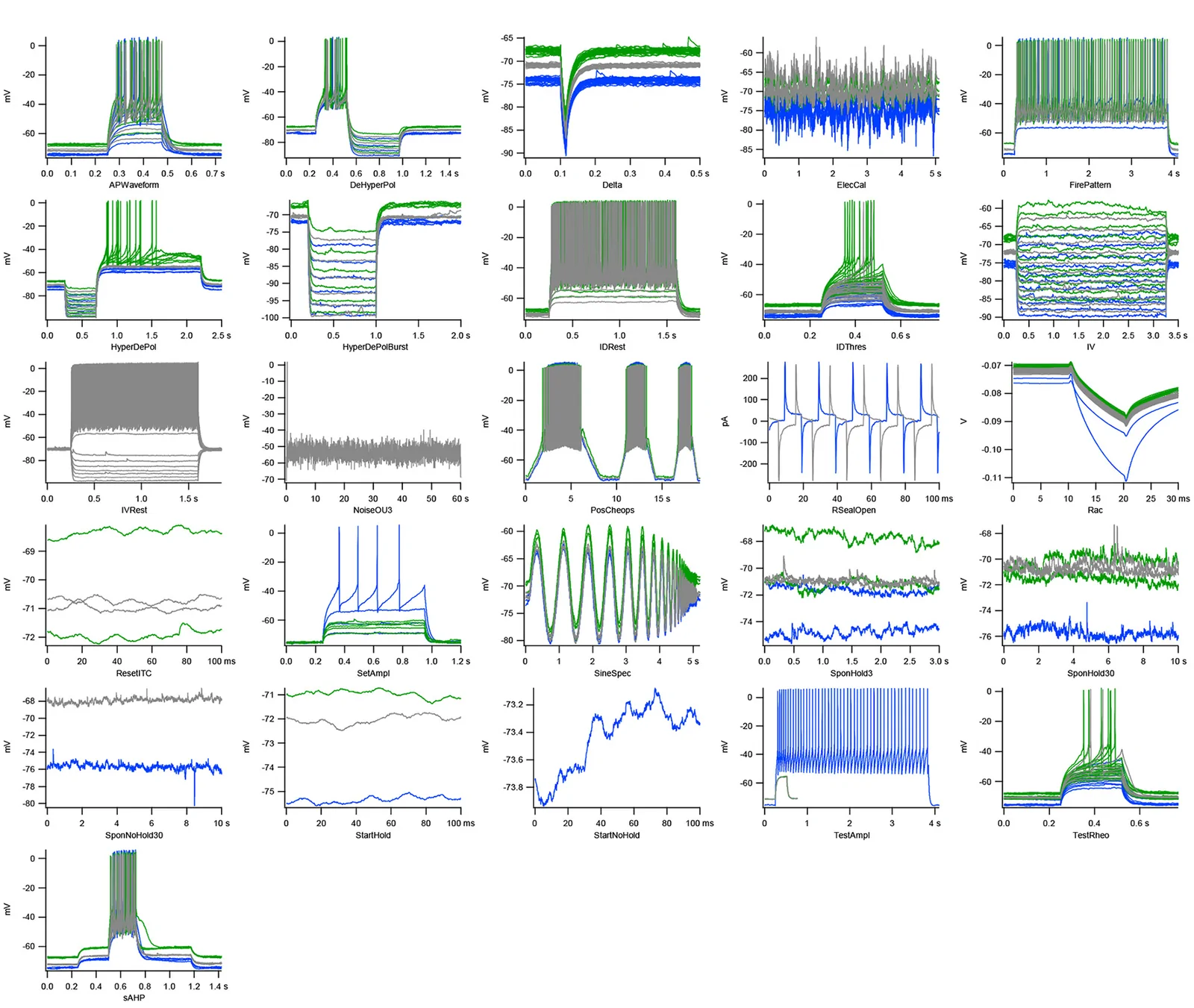
The full set of whole-cell current-clamp response traces from a single recording (cell MPG210604Th_A_1), drawn across the distinct stimulus protocols. Protocols aimed at measuring voltage responses were run at least two times, for internal consistency (each color is a full set repetition of a stimulus protocol). The stimuli set is aimed at capturing a broad set of different voltage responses in subthreshold, threshold, and suprathreshold conditions. Some recordings are for internal screening and tracing (e.g., TestAmpl, SartNoHold, SpontNoHold30), so they are performed either at the beginning and/or at the end of the trials. Some protocols screen a response through a voltage range so they have multiple steps (e.g., IV, IDRest, or APWaveform). Some protocols have triplicates, so they obtain a less noisy average trace for analysis (e.g., SineSpec). Membrane potentials are plotted in millivolts against time in seconds. For RSealOpen (voltage clamped at −70 mV with a ±5 mV step), recordings are in picoamps against time in milliseconds.

The composition of the recordings and their acquisition parameters are summarized in Figure 4.

**Figure 4:**
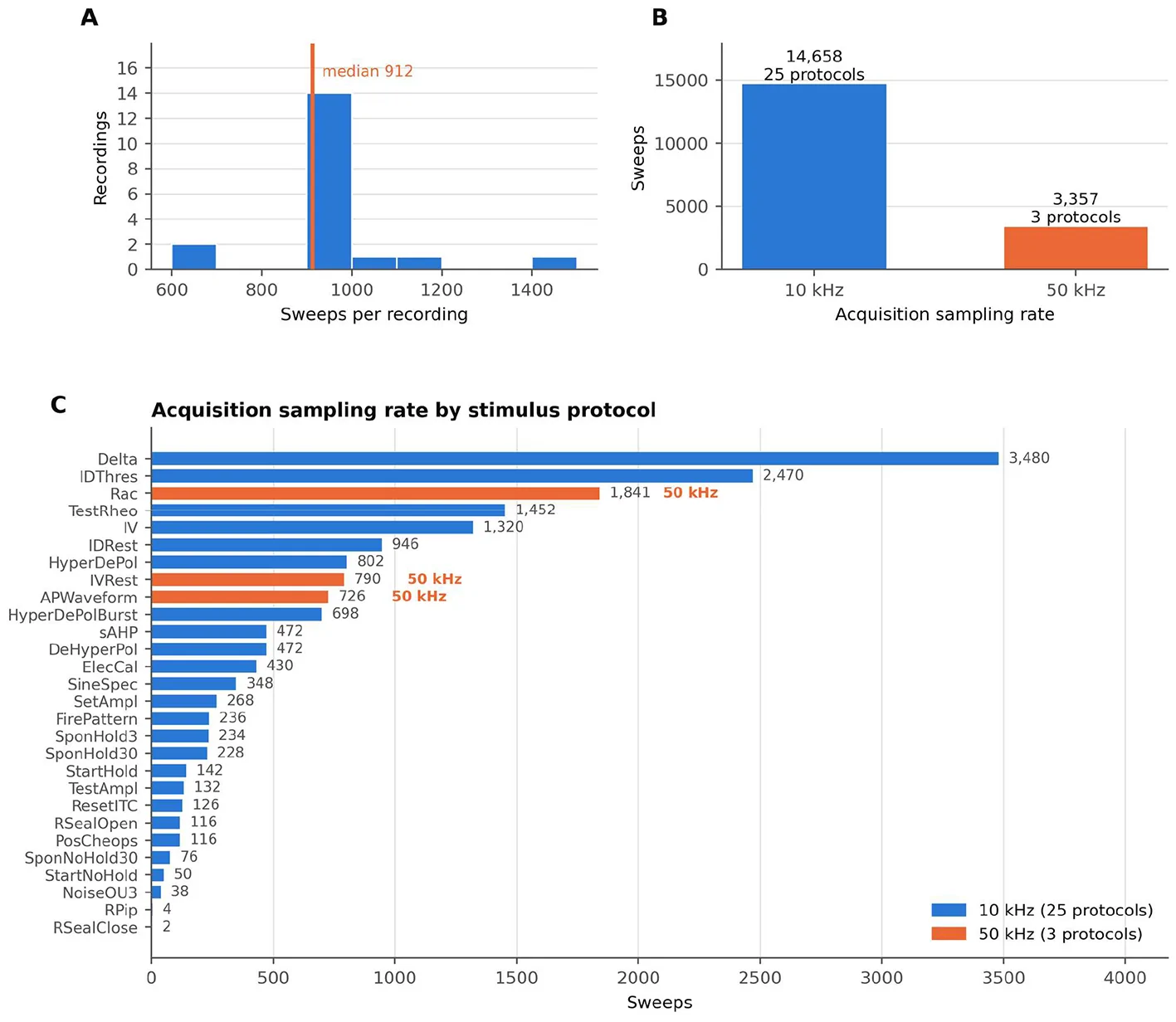
Recording composition and acquisition sampling rate. **(A)** Distribution of the number of sweeps per recording (median 912; range 658–1,494); the orange rule marks the median. **(B)** Total sweeps acquired at each sampling rate across all 18,015 sweeps: 14,658 sweeps from 25 protocols at 10 kHz, and 3,357 sweeps from 3 protocols at 50 kHz. **(C)** Acquisition sampling rate resolved by stimulus protocol. Each of the 28 protocols was acquired at a single rate—the mapping is exact, with no protocol split across rates. The three protocols recorded at 50 kHz are Rac (1,841 sweeps), IVRest (790), and APWaveform (726), each labeled on its bar; the remaining 25 protocols were recorded at 10 kHz.

## Discussion

### The value of the dataset

This resource provides standardized intrinsic electrophysiology for TRN neurons of juvenile mice, recorded under a single current-clamp stimulus battery applied identically across all cells. The uniform protocol and the preservation of raw signals in the community-standard NWB format—alongside the original Igor Pro sources—make the data directly suitable for characterizing TRN intrinsic properties, for cell-type and firing-phenotype analysis, for fitting and validating biophysically detailed single-neuron models (including the low-threshold rebound-burst behavior that defines this population), and for machine-learning studies of intrinsic electrophysiology. Indexing the full time series into tidy Parquet tables with a consistent session↔sweep↔sample join key lowers the barrier to querying and integrating the recordings at scale without opening every HDF5 file.

### The limitations of the dataset

The data reflect the LNMC recordings gathered by MPG and are not a population-representative survey: the cohort is 19 neurons from 13 male juvenile C57Bl/6J mice within a narrow developmental window, and no female animals are included. The recordings are electrophysiology only—no morphological reconstructions or histological imagery are distributed in this package. This release distributes the raw recorded signal and its acquisition metadata. Certain acquisition-level details are recorded only in the Igor Pro wave notes (protocol_def) rather than as parsed per-sweep stimulus amplitudes. Despite being just a small sample, it provides real, stable recordings of TRN neurons, with a thorough exploration of voltage responses within physiological ranges in a standardized manner.

### Opportunities for future research

Future extensions could add an extracted electrophysiological feature table (e.g., input resistance, rebound-burst latency and strength, firing rate, adaptation index, action-potential waveform metrics) computed directly from the NWB sweeps; parse per-sweep stimulus amplitudes from the wave-note protocol definitions to reconstruct full stimulus–response curves; broaden the cohort to include female animals and additional developmental windows; and pair the recordings with morphological reconstructions to build a cross-modal TRN resource. The standardized stimulus set also supports automated e-type classification and fitting and validation of biophysical models of TRN firing.

## Conclusion

This dataset provides a FAIR²-aligned, AI-ready resource of standardized intrinsic electrophysiology for neurons of the juvenile mouse TRN from the EPFL Laboratory of Neural Microcircuitry. With 19 whole-cell current-clamp recordings, 18,015 sweeps, ~400 million digitized samples, canonical NWB and Igor Pro assets, and tidy cross-linked tables, it supports reproducible analysis and machine-learning workflows on the intrinsic function of TRN neurons.

## Data Availability

The original contributions presented in the study are included in the article/supplementary material, further inquiries can be directed to the corresponding author.

## Author's note

Dataset curated from the EPFL LNMC project MPG archives; FAIR² packaging by SENSCIENCE. This document and the accompanying data package were prepared by the authors with assistance from the Frontiers FAIR² Data Management platform.

## References

[B1] Crandall S. R. Cruikshank S. J. Connors B. W. (2015). A corticothalamic switch: controlling the thalamus with dynamic synapses. Neuron 86, 768–782. doi: 10.1016/j.neuron.2015.03.040 PMC4425600 25913856

[B2] Halassa M. M. Acsády L. (2016). Thalamic inhibition: diverse sources, diverse scales. Trends Neurosci. 39, 680–693. doi: 10.1016/j.tins.2016.08.001 PMC5048590 27589879

[B3] MLCommons (2024). Croissant: A Metadata Format for ML-Ready Datasets. Available online at: https://github.com/mlcommons/croissant (Accessed July 03, 2026).

[B4] Open Data Commons (2017). ODC Attribution License (ODC-By v1.0). Available online at: https://opendatacommons.org/licenses/by/1-0/ (Accessed July 03, 2026).

[B5] Paxinos G. Franklin K. B. J. (2019). Paxinos and Franklin's The Mouse Brain in Stereotaxic Coordinates, 5th Edn (San Diego: Elsevier Academic Press).

[B6] Rübel O. Tritt A. Ly R. Dichter B. K. Ghosh S. Niu L. . (2022). The neurodata without borders ecosystem for neurophysiological data science. eLife 11:e78362. doi: 10.7554/eLife.78362 PMC9531949 36193886

[B7] Wilkinson M. D. Dumontier M. Aalbersberg I. J. Appleton G. Axton M. Baak A. . (2016). The FAIR Guiding Principles for scientific data management and stewardship. Sci. Data 3:160018. doi: 10.1038/sdata.2016.18 PMC4792175 26978244

